# Influence of Positive End-Expiratory Pressure on Myocardial Strain Assessed by Speckle Tracking Echocardiography in Mechanically Ventilated Patients

**DOI:** 10.1155/2013/918548

**Published:** 2013-08-28

**Authors:** Federico Franchi, Agnese Faltoni, Matteo Cameli, Luigi Muzzi, Matteo Lisi, Lucia Cubattoli, Sofia Cecchini, Sergio Mondillo, Bonizella Biagioli, Fabio Silvio Taccone, Sabino Scolletta

**Affiliations:** ^1^Department of Medical Biotechnologies, Unit of Intensive Care Medicine, University of Siena, Viale Bracci 10, 53100 Siena, Italy; ^2^Department of Medical Biotechnologies, Unit of Cardiology, University of Siena, Viale Bracci, 53100 Siena, Italy; ^3^Department of Intensive Care, Erasme University Hospital, Route de Lennik, 800-1070 Brussels, Belgium

## Abstract

*Purpose*. The effects of mechanical ventilation (MV) on speckle tracking echocardiography- (STE-)derived variables are not elucidated. The aim of the study was to evaluate the effects of positive end-expiratory pressure (PEEP) ventilation on 4-chamber longitudinal strain (LS) analysis by STE. *Methods*. We studied 20 patients admitted to a mixed intensive care unit who required intubation for MV and PEEP titration due to hypoxia. STE was performed at three times: (T1) PEEP = 5 cmH_2_O; (T2) PEEP = 10 cmH_2_O; and (T3) PEEP = 15 cmH_2_O. STE analysis was performed offline using a dedicated software (XStrain MyLab 70 Xvision, Esaote). *Results*. Left peak atrial-longitudinal strain (LS) was significantly reduced from T1 to T2 and from T2 to T3 (*P* < 0.05). Right peak atrial-LS and right ventricular-LS showed a significant reduction only at T3 (*P* < 0.05). Left ventricular-LS did not change significantly during titration of PEEP. Cardiac chambers' volumes showed a significant reduction at higher levels of PEEP (*P* < 0.05). *Conclusions*. We demonstrated for the first time that incremental PEEP affects myocardial strain values obtained with STE in intubated critically ill patients. Whenever performing STE in mechanically ventilated patients, care must be taken when PEEP is higher than 10 cmH_2_O to avoid misinterpreting data and making erroneous decisions.

## 1. Introduction

Echocardiography has become an indispensable diagnostic tool for the management of the critically ill patients in intensive care unit (ICU) [1]. Patients admitted to ICU often require positive-pressure mechanical ventilation (PPMV) with positive end-expiratory pressure (PEEP) due to severe hypoxia. Unfortunately, PPMV + PEEP may play a negative role in haemodynamics because it can lead to cardiac dysfunction by various mechanisms [2]. Accordingly, whenever focussing on echocardiographic parameters for the management of critically ill patients, physicians have gained experience to take into account the negative influence that mechanical ventilation (MV) may have on cardiac function [1, 3, 4].

The standard echocardiographic parameters are not always easy to achieve in critically ill patients, due to the dependence on the angle of insonation. Furthermore, acquiring some unusual echocardiographic parameters can rise some difficulties by noncardiologists and not experienced operators [5, 6].

Speckle tracking echocardiography (STE) is a new noninvasive ultrasound imaging technique that allows for an objective and quantitative evaluation of myocardial function, less dependent of the angle of insonation and of cardiac translational movements, compared to Doppler approaches [7–9]. STE allows an objective evaluation of four-chamber myocardial strain, which has been demonstrated to be a valuable marker of cardiac function and a good predictor of outcome [10–16]. In addition, the semiautomated nature of STE guarantees good intraobserver and interobserver reproducibility, which is a desirable characteristic in mixed ICU [8, 9].

To our knowledge, in intubated patients no studies have been performed to evaluate the changes of myocardial strain induced by mechanical ventilation. Thus, the influence of PPMV with high levels of PEEP on STE-derived variables remains to be elucidated.

The aim of the study was to evaluate the effects on the longitudinal strain (LS) of the four cardiac chambers at different levels of PEEP.

## 2. Materials and Methods

### 2.1. Study Population

We performed a prospective observational study at the mixed Intensive Care Unit (ICU) of the University Hospital of Siena. Approval from the institutional review board was obtained, along with written informed consent from patients or their legal representative. We enrolled 20 consecutive patients (male 8, mean age 64 ± 18), admitted to our ICU due to heterogeneous pathologies, who needed intubation for mechanical ventilation. Inclusion criteria were hypoxia requiring PEEP levels optimization, invasive arterial pressure monitoring, and age > 18 years. Exclusion criteria were the presence of active air leak (pneumothorax, subcutaneous emphysema, and pneumomediastinum) and chronic obstructive bronchopneumopathy, intracranial pressure > 20 mmHg, hemodynamic instability (defined as mean arterial pressure [MAP] < 70 mmHg and cardiac index [CI] < 2.0 L/min/m^2^), the presence of preexisting myocardial akinesia, not sinus rhythm, severe mitral or aortic regurgitation or stenosis and ascending aortic diseases, mitral stenosis, any prosthetic mitral and/or aortic valve, and an insufficient imaging quality of the endocardial border.

Patients were sedated with propofol 2% (0.5–2 mg/kg/hr) or midazolam (0.5–2 mg/kg/hr) and fentanyl (0.5–1 mcg/Kg/hr). All of them were equipped with a radial arterial catheter and a central venous catheter. Patients were ventilated on volume controlled mechanical ventilation (Servo *i*, Maquet critical care AB, Sweden).

### 2.2. Study Design

Standard echocardiography (MyLab 70 Xvision, Esaote) was performed by the same operator for each patient after having increased PEEP three times: T1 = 5 cmH_2_O, T2 = 10 cmH_2_O, and T3 = 15 cmH_2_O (according to our internal protocol) until reaching the best oxygenation with minimal negative hemodynamic effects. Measurements were performed after 5 minutes of stable mean arterial pressure and under haemodynamic steady-state conditions (about 10 minutes after having reached each level of PEEP). At each time of study a second operator measured the parameters of respiratory mechanics: intrinsic PEEP (PEEPi, with end-expiratory pause of 3 seconds), plateau pressure (Pplat), peak pressure (Ppeak), TV, RR, and static compliance of the respiratory system (Crs) were calculated using the standard formula. At the same time, a third operator estimated various hemodynamic parameters (heart rate, HR; stroke volume, SV; cardiac output, CO; and mean arterial pressure, MAP) using the pulse contour method MostCare (Vygon, Padua, Italy) [17–19]. STE analysis of the four cardiac chambers was performed offline using a dedicated software (XStrain MyLab 70 Xvision, Esaote).

### 2.3. Standard Echocardiography

Echocardiography was performed using a high-quality echocardiograph (MyLab 70 Xvision, Esaote). Patients were studied in the supine position. Bidimensional and Doppler measurements were made in accordance with current recommendations of the American Society of Echocardiography [20]. Left ventricular-ejection fraction (LV-EF) was measured using the modified biplane Simpson's rule [20]. The ratio between peak early (E) and late (A) diastolic LV filling velocities was used as standard indices of LV diastolic function [21]. M-mode measurements of mitral annular plane systolic excursion (MAPSE) were performed by placing the cursor perpendicular to the lateral site of the annulus; this was used as an index of LV longitudinal function [22]. The M-mode measurement of tricuspid annular plane systolic excursion (TAPSE) was calculated with the M-mode cursor aligned through the tricuspid annulus in the apical 4-chamber view; longitudinal displacement of the annulus toward the apex during systole was considered as an index of right ventricular (RV) systolic function [23].

### 2.4. Speckle Tracking Echocardiography (STE)

For speckle tracking analysis, apical four- and two-chamber and apical long-axis view images were obtained using conventional two-dimensional grayscale echocardiography, with a stable ECG recording. Particular attention was given to obtain an adequate gray scale image, allowing reliable delineation of myocardial tissue and extracardiac structures. For measurements, three consecutive heart cycles were recorded and averaged. The frame rate was set between 60 and 80 frames per second. These settings are recommended to combine temporal resolution with adequate spatial definition and to enhance the feasibility of the frame-to-frame tracking technique [24]. Recordings were processed using an acoustic-tracking software (XStrain MyLab 70 Xvision, Esaote), allowing offline semiautomated analysis of speckle-based strain [25, 26]. Briefly, endocardial surface is manually traced in apical views by a point-and-click approach. The software processes this track with the possibility of further manual correction of its shape and divides it into 6 segments of interest. Segments in which no adequate image quality is obtained can be rejected and excluded from the analysis. Finally, the software generates strain curves for each segment and gives the averaged values for longitudinal strain and time to peak longitudinal strain (TPLS).

Global longitudinal strain was defined by averaging longitudinal peak strain measured in apical 4- and 2-chamber and apical long-axis views.

The right ventricular-longitudinal strain (RV-LS) was calculated by averaging values observed in all RV segments [14].

Peak atrial longitudinal strain (PALS) was calculated by averaging values observed in all LA and RA segments (LA and RA global PALS, resp.) and by averaging values observed in 4- and 2-chamber views (4- and 2-chamber average PALSs). Care was taken to obtain accurate apical images using standard anatomic landmarks in each view and not foreshorten the atrial chambers, allowing a more reliable delineation of the atrial endocardial border. TPLS was also measured as the average of all 12 segments (global TPLS) and by separately averaging values observed in the two apical views (4- and 2-chamber average TPLSs). In patients in whom some segments were excluded because of the impossibility of achieving adequate tracking, ventricular-longitudinal strain, PALS, and TPLS were calculated by averaging values measured in the remaining segments.

### 2.5. Reproducibility

The reproducibility and the feasibility of STE measurements of 4-chamber longitudinal strain have been previously reported [15, 16].

### 2.6. Statistical Analysis

Statistical analysis was performed using the software SPSS 17.0 (Chicago, Inc, USA). To test the normal distribution of the data the Kolmogorov-Smirnov test was used. Statistical differences were verified by one-way ANOVA. All data are presented as mean ± standard deviation (SD) or number and percentage when appropriate.

In order to investigate the potential interrelation between the variables (the size of the four heart chambers, strain and stroke, volume), we performed the Pearson's correlation test for changes over time (Δ) of the variables. Changes of (Δ) in size, strain, and stroke volumes were calculated by subtracting the first from the third value (i.e., LV-EDV, left ventricular end diastolic volume at T3—LV-EDV at T1). Furthermore, to account for the effect of changes in size and strain values on stroke volume, patients were divided into two groups according to their reduction in SV (i.e., SV ≤ 15%, SV > 15%) after having increased PEEP from 5 to 15 cmH_2_O [27]. Finally, to track the relationship between these changes we plotted the Δ*s* into four quadrant plot graphs [18, 28]. A *P* value less than 0.05 was considered statistically significant.

## 3. Results

**Figure 1:**
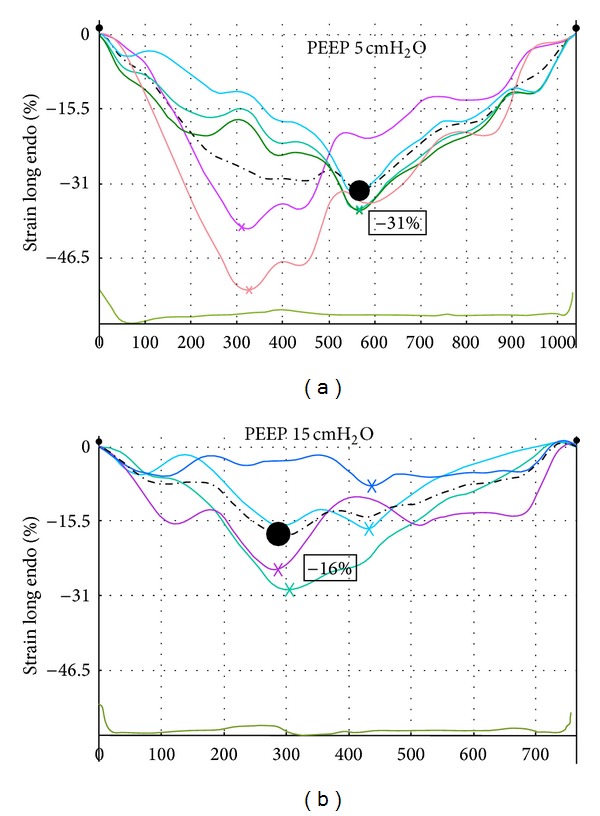
The figure shows an STE examination performed to evaluate the myocardial longitudinal strain (LS) of the right ventricle (RV) in a patient under study. (a) shows that in case of PEEP = 5 cmH_2_O the nadir point (white circle) upon the white dotted line is −28%. (b) shows that the same examination conducted by increasing PEEP at 15 cmH_2_O causes a reduction of the nadir point up to −10% due to the influence of increased PEEP on RV function. The dotted line represents the RV-LS value averaged over other lines.

**Figure 2:**
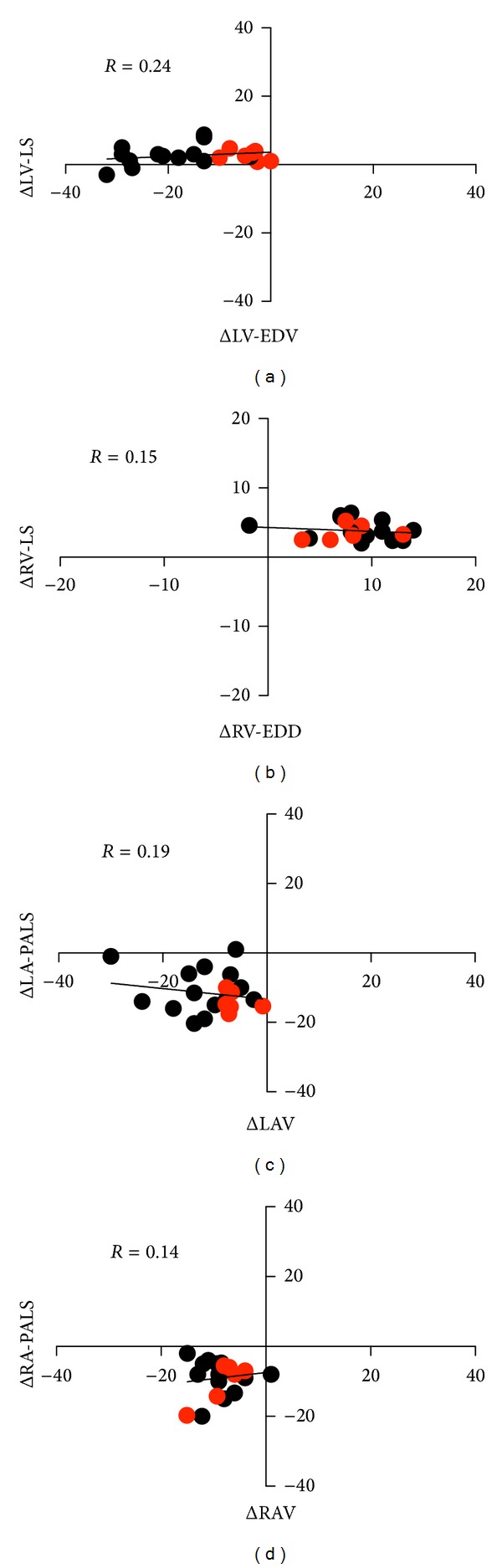
Four-quadrant trend plot for tracking the relationship in changes of size, strain values of the four cardiac chambers, and stroke volumes. (a) Left ventricle; (b) right ventricle; (c) left atrium; and (d) right atrium. There are no significant correlations between changes in strain values and size of the four cardiac chambers. Black dots indicate patients with a reduction in SV ≤ 15%; red dots indicate patients with a reduction in SV > 15%. Δ represents the changes in measures over time, obtained by subtracting the first measurement (at PEEP of 5 cmH_2_O) from the third (at PEEP of 15 cmH_2_O); LV-EDV; left ventricular end diastolic volume; LV-LS; left ventricular-longitudinal strain; RV-EDD; right ventricular end diastolic diameters; RV-LS; right ventricular-longitudinal strain; LAV; left atrial volume; LA-PALS; peak left atrial longitudinal strain; RAV; right atrium volume; and RA-PALS; peak right atrial longitudinal strain.

Patient characteristics are shown in Table 1. Hemodynamic and respiratory data are in Table 2. Pplat and Crs increased significantly from baseline (T1) to T3, that is when PEEP increased from 5 to 15 cmH_2_O (Table 2). SV showed a significant decrease from T1 to T3 (78.5 ± 18.8 mL versus 59.5 ± 14.2 mL; *P* < 0.05) and CO did the same (T1 = 5.6 ± 1.4 L/min versus T3 = 4.3 ± 0.8 L/min; *P* < 0.05). Cardiac chambers volumes (LV end diastolic volume and left and right atrial volumes) showed a significant reduction with incremental PEEP (Table 3). Conversely, right ventricular end diastolic diameter showed a significant increase from T1 to T2 and T3 (29.9 ± 5.9 mm, 38.0 ± 1.4 mm, and 40.0 ± 0.1 mm, at PEEP of 5, 10, and 15 cmH_2_O, resp.; *P* < 0.05) (Table 3). LV-EF, E/A ratio, TAPSE, and MAPSE did not change significantly (Table 3). LA-PALS was significantly reduced with incremental PEEP (40.2 ± 12.0% at T1, 35.9 ± 9.1 at T2, and 28.5 ± 7.9% at T3; *P* < 0.05) (Table 3). RA-PALS and RV-LS significantly decreased only at T3 (RA-PALS: 44.7 ± 48.5% at T1 versus 35.9 ± 10.7% at T3; RV-LS: −20.2 ± 2.1% at T1 versus −16.3 ± 1.2% at T3; *P* < 0.05) (Table 3, Figure 1). Conversely, LV-LS did not change significantly (Table 3). Comparison of the changes (Δ) in size and strain for the four chambers did not show significant correlation (*R* = 0.24, *R* = 0.15, *R* = 0.19, and *R* = 0.14 for LV, RV, LA, and RA, resp.; *P* > 0.05) (Figure 2). All patients had a reduction in SV with incremental PEEP. When PEEP changed from 5 to 15 cmH_2_O, the patients with an SV reduction lower than 15% (6 patients) showed a greater reduction in LV-EDV and LAV than those with an SV reduction > 15% (LV-EDV −4.3 ± 3.6 versus −17.3 ± 9.4, for patients with an SV reduction ≤ 15% and >15%, resp.; *P* = 0.05; LAV −6.2 ± 2.7 versus −13.7 ± 7.5 for patients with an SV reduction ≤ 15% and >15%, resp.; *P* = 0.05) (Table 4, Figure 2). Changes in four-chamber strain values between patients with a reduction in SV lower or greater than 15% were not significant (Table 4).

## 4. Discussion

The major finding of this study is a significant reduction of left atrial-, right atrial-, and right ventricular-longitudinal strain values during the increase of PEEP levels. Conversely, left ventricular-longitudinal strain values did not show significant changes. To our knowledge, this is the first study that evaluates the influence of different levels of PEEP on 4-chamber longitudinal strain obtained by STE in intubated patients.

The use of echocardiography is increasing in critically ill patients, for evaluating either the cardiac function or the potential negative effects of mechanical ventilation on the cardiovascular system. The latter is particularly important in patients with severe respiratory failure, such as acute respiratory distress syndrome (ARDS), in whom the usage of very high levels of PEEP is usual [1, 3, 4, 29].

Although physicians have gained familiarity with “rummy echocardiographic data” that are frequently observed in intubated patients ventilated with PEEP, some potential limitations with the use of echocardiography in critically ill patients still remain: firstly, there could be a certain difficulty to obtain some angle-dependent parameters in mechanically ventilated patients in the supine position [5, 6]; secondarily, there is a lack of reproducibility of different parameters (e.g., cardiac chambers volumes) due to the operator dependency [5, 6]. These drawbacks are even more critical in mixed ICU, because the operators are often noncardiologists and not extremely experienced with echocardiography [6]. In this view, STE due to its semiautomatic technology and its less dependence on the angle of insonation, with respect to the Doppler approaches, seems to be a promising method to study the cardiac function in patients admitted to mixed ICU [7–9].

STE is based on an analysis of the spatial dislocation (referred to as tracking) of speckles (defined as spots generated by the interaction between the ultrasound beam and myocardial fibers) on routine 2-dimensional sonograms [8]. By tracking the displacement of speckles during the cardiac cycle, speckle tracking echocardiography allows semiautomated elaboration of myocardial deformation (myocardial strain) in 3 spatial directions [8].

In general, STE may allow an evaluation of myocardial systolic and diastolic dynamics across a broad range of physiologic and pathologic conditions beyond traditional echocardiographic techniques. Indeed, valuable strain values have been obtained in pathologic conditions as hypertension, diabetes, heart failure, coronary artery disease, and cardiac dyssynchrony and after heart transplantation [10–16].

Although STE may have some advantages over standard echocardiography (e.g., it is less affected by the angle dependence), misleading interpretation of STE-derived data may occur in intubated patients, and the potential effects of PPMV + PEEP on such parameters have not yet been elucidated and codified. 

The mechanisms by which the PEEP alters the hemodynamic status of patients are complex and are also influenced by pathologies of the respiratory system (e.g., chronic obstructive bronchitis, ARDS). The main hemodynamic effect of PEEP is the impairment of cardiac function related to lung volume and intrathoracic pressure (ITP) changes [30]. Briefly, when looking at the right side of the heart, the PEEP determines a reduction of the venous return due to an increase in right atrial pressure secondary to increased ITP [30, 31]. Also, high levels of PEEP may result in pulmonary overdistension with an increase in pulmonary vascular resistance (and therefore of ventricular afterload) and left shift of the interventricular septum [30]. When looking at the left side of the heart, the PEEP causes a reduction of SV secondary to the shift of the interventricular septum and the increased pericardial pressure generated by the augmented ITP [30]. In addition, PEEP reduces LV afterload by increasing the pressure gradient between LV and aorta [28]. Notably, all the aforementioned hemodynamic effects of PEEP are strictly influenced by intravascular volume and the degree of pulmonary distension [30, 31].

A number of studies have focussed on how high PEEP values could influence traditional echocardiography measurements, [30, 32–34] but no data are available on the potential effects that mechanical ventilation might have on STE-derived data. In our study, for the first time it has been observed that LA-PALS was significantly reduced by PEEP. Also, RA-PALS and RV-LS showed a significant decrease at PEEP of 15 cmH_2_O. In other words, we found that the higher the PEEP, the lower the LA- and RA-PALS and RV-LS. Conversely, LV-LS did not change significantly with incremental PEEP (Table 3). The four quadrant plot graphs show that, for all cardiac chambers, changes in volume and strain were not correlated. In other words, incremental PEEP seemed not to affect simultaneously, similarly, and in a predictable manner the size and the strain of the four chambers (Figure 2, Table 4). 

When focussing on the left ventricle, it seemed that its reduction in size, combined with a more or less maintained strain, might explain the reduction in SV (Table 3, Figure 2(a)). In addiction, the patients whose reduction in SV was greater than >15% showed a significant reduction in LV-EDV but not in LV strain. Therefore, changes in LV contractility and pressure were unlikely (Table 4, Figure 2(a)). 

The right ventricle showed an increase in size and a decrease in strain but also a decrease in SV, likely indicating that there was also a change in RV pressure (Table 3, Figure 2(b)).

Finally, with incremental PEEP, the size and the strain of the atria were significantly reduced (Table 3, Figures 2(c) and 2(d)). Of note, left atrium showed the highest volume reduction, particularly in patients who exhibited a significant reduction of their SV (>15%) (Table 4, Figure 2(c)). Conversely, the changes in strain of the left atrium were similar between the two groups (Table 4, Figure 2(c)). Similarly the changes in size and strain of the right atrium were not significantly different between the two groups. Based on these findings, one can infer that the atria are more affected by the heart-lung interaction than the ventricles. Thus, the higher the PEEP, the greater the pressure and the lower the volume and the properties of deformation of the atria.

Unfortunately, as mentioned above, the lack of the literature in describing the changes of STE-derived variables induced by PEEP does not allow us to make any comparison with our findings, which need further confirmatory studies.

Global longitudinal strain has recently been validated as a quantitative index of global LV function, showing a good correlation with LV-EF [7, 8]. Similarly, longitudinal myocardial deformation by STE has been used to evaluate the strain of left and right atria and right ventricle [14–16]. Moreover, STE analysis was also performed in spontaneous breathing patients who were assisted with continuous positive airway pressure (CPAP) [35, 36]. In these cohorts, the longitudinal strain of the ventricles resulted in a prognostic marker of long-term efficacy of CPAP on ventricular function, but no data is reported on myocardial strain changes when PEEP increased. On the contrary, we have established the changes in longitudinal strain of the heart chambers determined by the direct application of incremental PEEP. 

Of note, our data show that incremental PEEP had no effect on LV-LS. This parameter is an indicator of LV contractility that can change in various pathological conditions (e.g., heart failure), and whose reduction occurs before deterioration of LV-EF [8]. 

Experimental studies have shown that although CO is affected by incremental PEEP many indices of LV contractility, such as LV-EF, end systolic indices, and dP/dt are not [37–39]. The reduction of CO during application of PEEP is therefore mainly due to the reduction of preload, while contractility is preserved. Our data are in line with these findings, as LV-LS, MAPSE, and LV-EF did not change despite increasing the PEEP up to 15 mmHg (Tables 3 and 4). Conversely, with incremental PEEP, LV-EDV was significantly reduced along with LA- and RA-LS (and volumes) indicating a reduction of preload (Tables 3 and 4). 

By increasing PEEP, RV-EDD increased significantly and RV-LS showed a significant reduction (Figure 1), although other indices of right ventricular function (e.g., TAPSE) did not change significantly (Tables 3 and 4). We can only speculate that this can be related to the higher sensitivity of RV-LS to PEEP changes than the other variables [40, 41].

Some limitations have to be taken into account whenever using STE in critically ill patients undergoing mechanical ventilation. Primarily, as for traditional echocardiography, an adequate apical view is required to get different data. However, some studies have shown that an apical view is feasible in the majority of intubated patients [6, 12, 42]. Secondarily, nonsinus rhythm can affect the reliability of STE, because there is the need of averaging 3 different chamber-view measurements to derive longitudinal strain [7, 8].

Some limitations of our study have to be addressed. The small sample size did not allow us to explore the changes of myocardial strain associated with PEEP in patients with very poor lung compliance. In addition, to confirm the hypothesis that volume status can influence the changes in cardiac chambers strain induced by high levels of PEEP, it would have been useful to perform a fluid challenge. However, the present paper is an observational study, and the decision of administrating fluids was based on physicians' judgment, according to the needs of the single patient. Finally, it would have been more informative having performed STE in the same patients after extubation, in order to assess how STE-derived variables would have changed by shifting mechanical ventilation to spontaneous breathing.

## 5. Conclusions

In critically ill patients undergoing mechanical ventilation with PEEP, LA-, RA-PALS, and RV-LS significantly decrease related to incremental PEEP. Conversely, LV-LS remains stable demonstrating that the reduction of CO that occurs with incremental PEEP is preload-related and not contractility-related. 

Whenever performing STE and interpreting the myocardial strain values in mechanically ventilated patients to guide therapy, the physician should be aware that high levels of PEEP might influence STE-derived variables at a different degree. Specifically, care must be taken when PEEP is higher than 10 cmH_2_O to avoid misinterpreting data and making erroneous decisions. Further studies are warranted to confirm our results in an independent cohort.

## Figures and Tables

**Table 1:** Characteristics of the 20 patients. Values are expressed as mean ± standard deviation or numbers and percentage.

	*n* = 20
Age (years)	64 ± 18
Gender (male/female)	8/12
Weight (Kg)	75 ± 12
Body surface area (m^2^)	1.8 ± 0.2
APACHE II	16 ± 6
Intracerebral hemorrhage	8
Encephalitis	2
Polytrauma	6
Cerebral ischemia	2
Sepsis	2

APACHE II: Acute Physiology and Chronic Health Evaluation II.

**Table 2:** Respiratory and hemodynamic profile of the patients. Values are expressed as mean ± standard deviation.

	PEEP 5 cmH_2_O (T1)	PEEP 10 cmH_2_O (T2)	PEEP 15 cmH_2_O (T3)
Ventilatory parameters			
TV (mL)	498 ± 98	477 ± 68	465 ± 78
Pplat (cmH_2_O)	24.5 ± 2.9	29.5 ± 2.8*	34.6 ± 2.8^#^
C.rs (mL/cmH_2_O)	30.9 ± 18.5	33.2 ± 15.6	34.2 ± 15.0^#^
Hemodynamic parameters			
MAP (mmHg)	87 ± 19	86 ± 15	78 ± 14
HR (beats per minute)	74 ± 21	74 ± 21	75 ± 21
CO (L/min)	5.6 ± 1.4	4.9 ± 0.9*	4.3 ± 0.8^∗#^
SV (mL)	78 ± 19	70 ± 13*	59 ± 14^∗#^

TV: tidal volume; Pplat: plateau pressure; C.rs: static compliance of the respiratory system; MAP: mean arterial pressure; HR: heart rate; CO: cardiac output; SV: stroke volume. *P < 0.05 between the three times (T1, T2, and T3) of the study; ^#^ *P* < 0.05 between the first (T1) and the third time (T3) of the study.

**Table 3:** Standard echocardiographic profile of the patients together with speckle tracking echocardiography (STE) data. Values are expressed as mean ± standard deviation.

Parameter	PEEP 5 cmH_2_O (T1)	PEEP 10 cmH_2_O (T2)	PEEP 15 cmH_2_O (T3)
Standard echocardiography			
Mitral E/A	1.1 ± 0.4	1.1 ± 0.5	0.9 ± 0.3
PAPs	32.8 ± 12.1	38.3 ± 2.9	41.5 ± 7.5
LV-EF (%)	54.6 ± 6.6	52.7 ± 5.7	50.4 ± 6.2
LV-EDD (mm)	45.9 ± 7.6	45.0 ± 5.7	42.5 ± 4.94^#^
RV-EDD (mm)	29.9 ± 5.9	38.0 ± 1.4*	39.0 ± 0.1^#^
LV-EDV (mL)	88.7 ± 18.2	85.5 ± 23.2*	78.9 ± 22.5^∗#^
LAV (mL)	49.9 ± 15.3	41.2 ± 11.2*	40.0 ± 8.5^∗#^
LAV/BSA ratio (mL/m^2^)	30.5 ± 8.5	24.6 ± 6.8*	21.6 ± 5.1^∗#^
RAV (mL)	41.9 ± 8.8	37.8 ± 11.0	33.6 ± 9.8^#^
MAPSE (mm)	15.1 ± 2.6	14.2 ± 2.5	14.7 ± 1.8
TAPSE (mm)	21.7 ± 2.6	21.8 ± 2.2	19.7 ± 2.4
Speckle tracking echocardiography			
LV-LS (%)	−18.3 ± 2.6	−16.8 ± 2.4	−15.2 ± 3.0
LV TPLS (msec)	390.0 ± 36.7	407.2 ± 52.9	393.4 ± 61.9
RV-LS (%)	−20.2 ± 2.1	−19.9 ± 2.9	−16.3 ± 1.2^#^
RV TPLS (msec)	377.4 ± 37.5	403.9 ± 53.4	416.7 ± 46.4
LA-PALS (%)	40.2 ± 12.0	35.9 ± 9.1*	28.5 ± 7.9^∗#^
LA TPLS (msec)	381.6 ± 33.6	394.7 ± 49.4	372.3 ± 62.2
RA-PALS (%)	44.7 ± 8.5	41.5 ± 12.4	35.9 ± 10.7^#^
RA TPLS (msec)	388.3 ± 48.5	409.0 ± 56.4	392.4 ± 65.2

E/A ratio: E wave velocity/A wave velocity ratio; PAPs: systolic pulmonary arterial pressure; LV-EF%: left ventricular ejection fraction; LV-EDD: left ventricular end diastolic diameters; RV-EDD: right ventricular end diastolic diameters; LV-EDV: left ventricular end diastolic volume; LAV: left atrial volume; BSA: body surface area; RAV: right atrium volume; MAPSE: mitral annular plane systolic excursion; TAPSE: tricuspidal annular plane systolic excursion; LV-LS: left ventricular longitudinal strain; LV TPLS: left ventricular time to peak longitudinal strain; RV-LS: right ventricular longitudinal strain; RV TPLS: right ventricular time to peak longitudinal strain; LA-PALS: peak left atrial longitudinal strain; LA TPLS time-to-peak left atrial longitudinal strain; RA-PALS: peak right atrial longitudinal strain; RA TPLS: time-to-peak right atrial longitudinal strain. *P < 0.05 between the three times (T1, T2, and T3) of the study; ^#^ *P* < 0.05 between the first (T1) and the third times (T3) of the study.

**Table 4:** Univariate analysis between patients with a stroke volume reduction (Δ) lower and greater than 15%. Values are expressed as mean ± standard deviation.

Parameter	Δ SV ≤ 15% (*N* = 6)	Δ SV > 15% (*N* = 14)	*P*
Δ LV-EDV (mL)	−4.3 ± 3.6	−17.3 ± 9.4	<0.05
Δ LV-LS (%)	3.0 ± 1.4	2.6 ± 3.1	0.79
Δ RV-EDD (mm)	9.5 ± 2.6	9.1 ± 4.3	0.83
Δ RV-LS (%)	3.9 ± 1.3	3.7 ± 1.4	0.89
Δ LAV ( mL)	−6.2 ± 2.7	−13.7 ± 7.5	<0.05
Δ LA-PALS (%)	−12.2 ± 3.7	−11.5 ± 6.6	0.80
Δ RAV (mL)	−8.7 ± 4.1	−8.7 ± 3.9	0.97
Δ RA-PALS (%)	−11.0 ± 6.9	−8.1 ± 3.9	0.24

Δ: changes in measures over time, obtained by subtracting the first measurement (at PEEP of 5 cmH_2_O) from the third (at PEEP of 15 cmH_2_O); LV-EDV: left ventricular end diastolic volume; LV-LS: left ventricular longitudinal strain; RV-EDD: right ventricular end diastolic diameters; RV-LS: right ventricular longitudinal strain; LAV: left atrial volume; LA-PALS: peak left atrial longitudinal strain; RAV: right atrium volume; RA-PALS: peak right atrial longitudinal strain.
